# Causal associations of genetically predicted gut microbiota and blood metabolites with inflammatory states and risk of infections: a Mendelian randomization analysis

**DOI:** 10.3389/fmicb.2024.1342653

**Published:** 2024-03-22

**Authors:** Yingjian Liu, Qian Zhu, Gongjie Guo, Zhipeng Xie, Senlin Li, Chengyang Lai, Yonglin Wu, Liansheng Wang, Shilong Zhong

**Affiliations:** ^1^Department of Pharmacy, Guangdong Provincial People's Hospital, Guangdong Academy of Medical Sciences, Southern Medical University, Guangzhou, Guangdong, China; ^2^Department of Neurosurgery, Huizhou Third People's Hospital, Guangzhou Medical University, Huizhou, Guangdong, China; ^3^School of Medicine, South China University of Technology, Guangzhou, Guangdong, China; ^4^Guangdong Cardiovascular Institute, Guangdong Provincial People's Hospital (Guangdong Academy of Medical Sciences), Southern Medical University, Guangzhou, China

**Keywords:** gut microbiome, infection, inflammation, Mendelian randomization analysis, metabolome

## Abstract

**Background:**

Inflammation serves as a key pathologic mediator in the progression of infections and various diseases, involving significant alterations in the gut microbiome and metabolism. This study aims to probe into the potential causal relationships between gut microbial taxa and human blood metabolites with various serum inflammatory markers (CRP, SAA1, IL-6, TNF-α, WBC, and GlycA) and the risks of seven common infections (gastrointestinal infections, dysentery, pneumonia, bacterial pneumonia, bronchopneumonia and lung abscess, pneumococcal pneumonia, and urinary tract infections).

**Methods:**

Two-sample Mendelian randomization (MR) analysis was performed using inverse variance weighted (IVW), maximum likelihood, MR-Egger, weighted median, and MR-PRESSO.

**Results:**

After adding other MR models and sensitivity analyses, genus *Roseburia* was simultaneously associated adversely with CRP (Beta _IVW_ = −0.040) and SAA1 (Beta _IVW_ = −0.280), and family *Bifidobacteriaceae* was negatively associated with both CRP (Beta _IVW_ = −0.034) and pneumonia risk (Beta _IVW_ = −0.391). After correction by *FDR*, only glutaroyl carnitine remained significantly associated with elevated CRP levels (Beta _IVW_ = 0.112). Additionally, threonine (Beta _IVW_ = 0.200) and 1-heptadecanoylglycerophosphocholine (Beta _IVW_ = −0.246) were found to be significantly associated with WBC levels. Three metabolites showed similar causal effects on different inflammatory markers or infectious phenotypes, stearidonate (18:4n3) was negatively related to SAA1 and urinary tract infections, and 5-oxoproline contributed to elevated IL-6 and SAA1 levels. In addition, 7-methylguanine showed a positive correlation with dysentery and bacterial pneumonia.

**Conclusion:**

This study provides novel evidence confirming the causal effects of the gut microbiome and the plasma metabolite profile on inflammation and the risk of infection. These potential molecular alterations may aid in the development of new targets for the intervention and management of disorders associated with inflammation and infections.

## Introduction

The inflammatory response is a vital component of immunity and acts as a critical mechanism against damage and infection. However, inflammation will become harmful when it loses control, spreads becomes systemic, or lasts for extended periods and becomes chronic. Inflammation can cause cellular injury, tissue destruction, cancer, organ failure, and death (Wang and Ma, 2008; Greten and Grivennikov, 2019), playing a key role in the pathology of various diseases. Infection represents a local tissue and systemic inflammatory response caused by pathogens invading the human body. Gastrointestinal infections (GI), pneumonia, and urinary tract infections (UTI) are common infections causing hospitalization and death (Collaborators, 2017). Therefore, it is essential to prevent and manage inflammation and infectious disorders (including their subtypes) with appropriate intervention strategies.

Systemic inflammation and infection are characterized by an increased release of cytokines and acute-phase proteins (APPs), as well as changes in the components of the blood (Slaats et al., 2016; Liu et al., 2017). Some of the most routinely used markers for inflammation and infection in clinical practice (Dos Anjos and Grotto, 2010; Menzel et al., 2021; Yin and Mo, 2022) include cytokines (e.g., interleukin-6 [IL-6] and tumor necrosis factor-alpha [TNF-α]), APPs (e.g., C-reactive protein [CRP] and serum amyloid a [SAA]), and blood cell counts (e.g., white blood cell count [WBC]), representing several components of the inflammatory process. Glycoprotein acetylation (GlycA) is a novel biomarker of systemic inflammation and cardiovascular disease (Connelly et al., 2017), reflecting both increased glycan complexity and circulating APPs. Elevated levels of these markers in the blood can be early signs of health effects (Qu et al., 2015; Zacho et al., 2016; Yin and Mo, 2022) and are clinically useful in tracing and detecting inflammatory severity and infectious risks, diagnosing, and following-up on diseases (Ponti et al., 2020; Menzel et al., 2021). However, most of the molecular mechanisms underlying inter-individual variation in systemic inflammation and the infectious risks remain to be illustrated.

It has been revealed that certain environmental and lifestyle factors can promote systemic inflammation, thereby contributing to disease development, severe disability, and mortality (Furman et al., 2019). The metabolome defines metabolic perturbations resulting from the interplay between the genome and environmental factors, representing an immediate host response to environmental exposures and pathological processes (Kaddurah-Daouk and Krishnan, 2009). Prior metabolomic studies have pinpointed specific metabolic changes associated with the urea cycle and oxidative stress, which correlate with inflammatory markers in healthy individuals (Pietzner et al., 2017) and rheumatoid arthritis patients (Jutley et al., 2021). Importantly, metabolomics was also widely applied to identify infection biomarkers (Araújo et al., 2022). Moreover, microbiota balance has a powerful regulatory effect on the human immune and metabolic systems (Samuelson et al., 2015). The gut microbiota, including its composition and metabolites such as lipopolysaccharides, bile acids, and short-chain fatty acids, also play important roles in inflammation development, significantly affecting host health (Al Bander et al., 2020; Tilg et al., 2020). At present, probiotics and micronutrients have been demonstrated to modulate some immune and inflammatory biomarkers and reduce the risk and severity of gastrointestinal and respiratory infections (Calder et al., 2022). Thus, the gut microbiota and metabolic signature are closely related to the host's immune-inflammatory response, potentially influencing the susceptibility to infections.

While research on the gut microbiome and blood metabolites is growing, the causal relationships between these factors and inflammation as well as the risk of infection remain poorly understood. Mendelian randomization (MR) is a powerful tool for inferring causal effects between exposures and outcomes (Davies et al., 2018). By leveraging genetic variants associated with the exposure, MR helps overcome the limitations of traditional observational studies, such as confounding and reverse causation. Therefore, we employed a two-sample MR approach to investigate the causal effects of 195 gut bacterial traits and 529 (290 annotated) human blood metabolites on inflammation and the risk of infections, aiming to gain deeper molecular insights. Our findings shed new light on the intricate relationships between the gut microbiome, blood metabolites, and inflammation and the risk of infections, which may have significant implications for preventing and treating inflammatory symptoms and infectious diseases.

## Materials and methods

**Figure 1:**
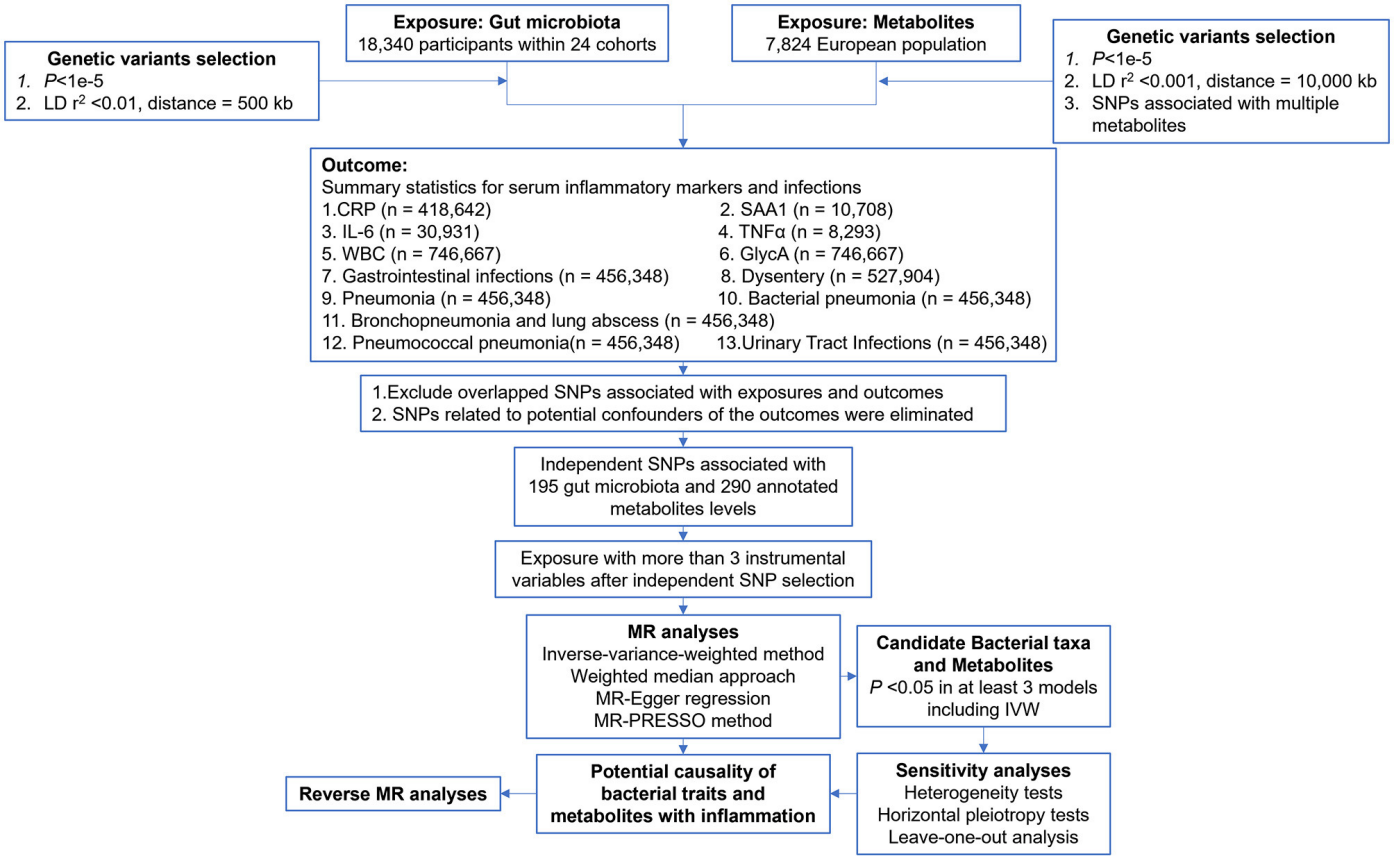
Flowchart of the MR analysis.

The study flow is depicted in Figure 1. A two-sample MR analysis was employed to assess the potential causal relationship between gut microbiota composition, blood metabolite levels, and nine outcomes. The MR analysis is based on the following three assumptions to avoid the causal estimates from being biased: (1) instrumental variables (IVs) are strongly associated with the exposure; (2) IVs are not correlated with the confounders; and (3) IVs can only affect the outcome through the exposure.

### Data source

The genetic predictors of human gut microbiome composition were obtained from a large-scale, multi-ethnic meta-analysis of microbiome-based GWAS, the MiBioGen study (Kurilshikov et al., 2021). This meta-analysis included 18,340 participants from 24 cohorts. Microbial abundance was based on 16S rRNA gene data, and the use of the direct taxonomic classification of reads, along with an up-to-date reference database, allowed for good concordance of taxonomic composition across domains and a higher mapping rate. After quality control (including duplication of the family *Bifidobacteriaceae* with the order *Bifidobacteriales* in the results of the GWAS, and deletion of larger taxonomic units of bacteria), a total of 195 microbial genera (119 genera, 32 families, 19 orders, 16 classes, and 9 phyla) were retained. Supplementary Table S1 provides a comprehensive list of all datasets used.

The metabolite GWAS dataset used in this study includes the largest meta-GWAS data to date, as published in Nature Genetics in 2014 by Shin et al. (2014). The dataset consists of a meta-analysis of seven European populations, comprising a total of 7,824 individuals of European ancestry. Following rigorous quality control procedures, 290 annotated blood metabolites (Supplementary Table S2) were included in the genome-wide association analysis.

CRP: The GWAS result of serum CRP levels was conducted within a large, population-based cohort of half a million individuals in the United Kingdom, aged between 40 and 69 years (Bycroft et al., 2018). Han et al. (2020) accessed serum CRP levels for 469,881 individuals from the 2019 serum biochemistry data release. The researchers calculated the mean serum CRP levels for individuals who had undergone two assessments. Prior to the GWAS analysis, a rank-based inverse normal transformation was applied to serum CRP levels to ensure a normal data distribution.

SAA1: A study integrates large-scale genomic and aptamer-based plasma proteomic data from 10,708 individuals prior to any SARS-CoV-2 infection or COVID-19 (Pietzner et al., 2020).

IL-6: The GWAS data for IL-6 is obtained from a study encompassing 90 cardiovascular proteins, involving more than 30,000 individuals from 15 distinct studies (Folkersen et al., 2020).

TNF-α: The TNF-α GWAS results were obtained from a GWAS meta-analysis that investigated the circulating concentrations of 41 cytokines, involving up to 8,293 Finnish individuals from three independent population cohorts (Ahola-Olli et al., 2017).

WBC: The WBC data are sourced from an extensive study providing genetic insights into blood cells across diverse populations, including 184,535 individuals of non-European ancestry, from a total of 746,667 individuals across five global populations (Chen et al., 2020).

GlycA: The GlycA GWAS meta-analysis was based on data from 249 metabolites quantified using nuclear magnetic resonance spectroscopy (NMR) in the UKB by Nightingale Health in 2020. Data can be accessed at https://gwas.mrcieu.ac.uk/datasets/met-d-GlycA/.

GWAS meta-analyses for GI (Phecode:008), pneumonia (Phecode:480), bacterial pneumonia (BP) (Phecode:480.1), bronchopneumonia, and lung abscess (BLA) (Phecode:480.5), pneumococcal pneumonia (PP) (Phecode:480.11), and UTI (Phecode:591) were derived using the GLMM-based GWA tool, fastGWA-GLMM. Application to UKB data included 456,348 individuals, 11,842,647 variants, and 2,989 binary traits (Jiang et al., 2021a). The meta-GWAS data for dysentery (Phecode:008.5) comes from meta-analysis across populations in three cohorts, namely, UKB, BioBank Japan, and FinnGen (Sakaue et al., 2021). All patients' diseases are systematically defined using the phecode framework; each phenotype (“phecode”) has defined case, control, and exclusion criteria (Denny et al., 2013).

### Selection of IVs

Additional quality control steps were implemented to select candidate IVs. Candidate IVs from the GWAS meta-results of gut microbiota and metabolites were included using a relatively relaxed standard of *P* < 1e^−5^ (Sanna et al., 2019). Considering that single nucleotide polymorphism (SNP) variations may affect several metabolites in the same pathway, thus violating the assumptions of the MR, a restrictive SNP selection is applied to metabolite research, excluding those that are significantly associated with more than two metabolites (Hwang et al., 2019). Genomic samples from the 1,000 Genomes Project (EUR) served as a linkage disequilibrium (LD) reference panel, and independent SNPs for gut bacteria and metabolites were retained using standards of R^2^ < 0.001, window size = 10,000 kb and R^2^ < 0.01, window size = 500 kb, respectively. The F-statistic, typically used to assess the strength of the correlation between IV and exposure, was calculated using the formula 1: F = R^2^ (n-k-1)/k(1-R^2^), where R^2^ is the variance of exposure explained by the selected SNP, n is the sample size, and k is the number of IVs included. IVs with an F-statistic of < 10 were considered weak and were excluded (Pierce et al., 2011). For metabolites, we used formula 2: F = (PVE(n-k-1))/(1-PVE) k, where PVE is the proportion of exposure variance for the selected IV. By setting k equal to 1, the PVE for each IV was calculated using the formula 3:

PVE = (2^*^β^2*^MAF^*^(1-MAF))/[(2^*^β^2*^MAF^*^(1-MAF)+2^*^Se^2*^ n^*^MAF^*^(1-MAF)].

Since the gut microbiota SNPs did not provide minor allele frequencies, R^2^ was estimated directly using the “get_r_from_pn” function from the “TwoSampleMR” package (Hemani et al., 2017, 2018) and then using formula 1 to calculate the F-statistic. Finally, horizontal pleiotropic effects, i.e., confounding effects caused by other diseases, may violate the second assumption in MR analysis (SNP is unrelated to outcome). Once these were detected, the associated IVs were removed. The MR-PRESSO test was used to detect potential horizontal pleiotropy (Verbanck et al., 2018). We mitigated the influence of pleiotropy by removing outliers, specifically SNPs with a global test *P-*value of < 0.05 in the MR-PRESSO test. After removing these outliers, we performed a re-analysis to ensure the accuracy of our results.

### Removing confounders

In adherence to the independence assumption of MR, we utilized PhenoScanner V2 to identify potential confounders for each outcome (Kamat et al., 2019). Our search parameters were the following. CRP (Luan and Yao, 2018): coronary heart disease, diabetes, Alzheimer's, Parkinson's, stroke, and macular degeneration. SAA1 (Sack, 2018): lipid profiles, atherosclerosis, cholesterol, and cancer. IL-6 (Tanaka et al., 2014): autoimmunity, chronic inflammatory diseases, inflammatory myopathies, juvenile idiopathic arthritis, rheumatoid arthritis, systemic sclerosis, and Castleman disease. TNF-α (Locksley et al., 2001; Brynskov et al., 2002; Swardfager et al., 2010): Alzheimer's, diabetes mellitus, inflammatory bowel disease, major depression, obesity, and psoriasis. WBC (Hasegawa et al., 2002): CAD and leukemia. GlycA (Connelly et al., 2017): body mass index, colorectal cancer, coronary artery disease, psoriasis, and rheumatoid arthritis. GI, dysentery (Lamps, 2009): salmonella, *E. coli*, giardiasis, cryptosporidiosis, viruses. Pneumonia, BP, BLA, PP (https://www.nhlbi.nih.gov/health/pneumonia/causes, accessed August 30, 2023): COPD, dementia, diabetes, heart failure, HIV, kidney diseases, Parkinson's, pregnancy, smoking, and stroke. UTI (Medina and Castillo-Pino, 2019): diabetes mellitus and viral infections. Potential confounders were subsequently excluded from the main MR analysis.

### MR analysis

The focus was on two relationships: between gut bacteria and serum inflammatory factors and the risks of infections and between blood metabolites and serum inflammatory factors and the risks of infections. This study is reported following the Strengthening the Reporting of Observational Studies in Epidemiology Using Mendelian Randomization guidelines (STROBE-MR, S1 Checklist) (Supplementary material 1). First, following the criteria mentioned earlier, we sequentially excluded the IVs with LD and confounders. Second, IVs with F-statistics < 10 were excluded. Third, after rigorously screening quality IVs, we explored potential causal relationships using two-sample MR with at least three IVs. Fourth, the following five methods were used to assess these associations: the inverse variance weighted (IVW) test (Burgess et al., 2013), the weighted median estimator (WME) (Pierce and Burgess, 2013), the maximum likelihood estimator (MLE) (Pierce and Burgess, 2013), the weighted mode-based estimator (Hartwig et al., 2017), and MR-Egger regression (Bowden et al., 2015). For multiple hypothesis testing, the false discovery rate (*FDR*) calculation for gut microbiota was performed at different taxonomic levels, while for metabolites, it was performed among the 290 annotated metabolites. A relationship was considered potentially causal if it was statistically significant in at least three of these methods, including the IVW method (Liu et al., 2022; Guo et al., 2023). Special attention was given to the IVW method because of its robustness to MR analysis. Fifth, a series of sensitivity analyses were used to ensure the robustness of our results. Finally, we performed bidirectional MR analysis on significant results to ensure the validity of the results and to avoid confusion in the causal interpretation.

### Sensitivity analysis

We used sensitivity analysis to ensure the robustness of our results and to identify potential biases such as pleiotropy and data heterogeneity. We also tested whether a particular instrumental variable significantly influenced the outcome variable. Our sensitivity analysis included a pleiotropy test, a heterogeneity test, and a leave-one-out method. For the pleiotropy test, we used the MR-PRESSO method and assumed that the horizontal pleiotropy of the IVs would not significantly affect the causal inference if the absolute value of the intercept was < 0.1 and the corresponding *P-*value was > 0.05. The heterogeneity test was used to identify differences between different IVs. *P-*value = 0.05 is the threshold value. The leave-one-out method allowed us to assess whether the MR estimate was driven or biased by a single SNP with a particularly large pleiotropic effect. This effect was re-estimated by sequentially removing one SNP at a time.

### Software

For data cleaning and structuring, we used Jupyter Notebook in Python (version 3.0). To perform the MR analysis, we used R (version 4.2.1) and the “TwoSampleMR” package.

## Results

### Selection of IVs

The characteristics of the selected IVs for each gut microbiota are listed in Supplementary Table S3. After conducting LD analyses and eliminating confounding variables, the ultimate counts of IVs for CRP, SAA1, IL-6, TNF-α, WBC, GlycA, GI, dysentery, pneumonia, BP, BLA, PP, and UTI are 1,594, 1,959, 1,021, 1,911, 1,597, 1,871, 1,870, 1,965, 1,876, 1,875, 1,864, 1,877, and 1,888, respectively. These IVs will be employed for subsequent MR analyses. Bacterial traits with fewer than three IVs were excluded.

Supplementary Table S4 provides the details of the selected IVs for each metabolite. Of the 56,147 SNPs significantly correlated with 290 blood metabolites (*P* < 1 × 10^−5^), 23,198 SNPs linked to at least two metabolites were excluded. After performing LD analyses and accounting for confounding factors, the instrumental variable counts for CRP, SAA1, IL-6, TNF-α, WBC, GlycA, GI, dysentery, pneumonia, BP, BLA, PP, and UTI are 2,879, 3,517, 2,619, 4,416, 3,031, 4,857, 5,095, 5,125, 5,066, 5,042, 5,068, 5,056, and 5,025, respectively. These instruments will be used in subsequent MR analyses, with the exclusion of metabolites having < 3 IVs.

In all analyses of serum inflammatory markers, the F-statistics of the IVs were >10, indicating less possibility of weak instrument bias.

### Two-sample MR analysis of gut microbiota on inflammation and risk of infections

We first used the IVW method as the primary analysis to evaluate the causal relationships between gut microbiota, serum inflammatory markers, and seven risks of infection (Supplementary Table S5). A total of 23 (including 14 genera), 9 (6 genera), 3 (2 genera), 8 (2 genera), 27 (16 genera), and 9 (6 genera) bacterial traits were suggested as significant for CRP, SAA1, IL-6, TNF-α, WBC, and GlycA (*P*_IVW_ < 0.05). Besides, 9 (3 genera), 6 (3 genera), 7 (2 genera), 9 (2 genera), 12 (6 genera), 5 (4 genera), and 8 (3 genera) bacterial traits were associated with risks of GI, dysentery, pneumonia, BP, BLA, PP, and UTI. Then, the other four MR methods, namely, MR-Egger, maximum likelihood, weighted mode-based estimator, and weighted median-based estimator, were added to further evaluate the causal estimates. The full results of all MR methods are presented in Supplementary Table S6. There are 7, 3, 1, 2, 9, and 1 suggestive causal associations detected for CRP, SAA1, IL-6, TNF-α, WBC, and GlycA across at least three MR methods (including IVW, *P* < 0.05). Additionally, 3, 2, 1, 4, 0, 1, and 1 associations were confirmed for the risks of GI, dysentery, pneumonia, BP, BLA, PP, and UTI using at least three MR methods. We next performed a heterogeneity and horizontal pleiotropy test to assess the robustness of the above suggestive associations (Supplementary Table S7). The above suggestive associations, where the intercept of the MR-Egger regression approached 0 and the *p*-values of both the MR-Egger and MR-PRESSO global tests were >0.05, indicating no evidence of horizontal pleiotropy, were retained.

**Figure 2:**
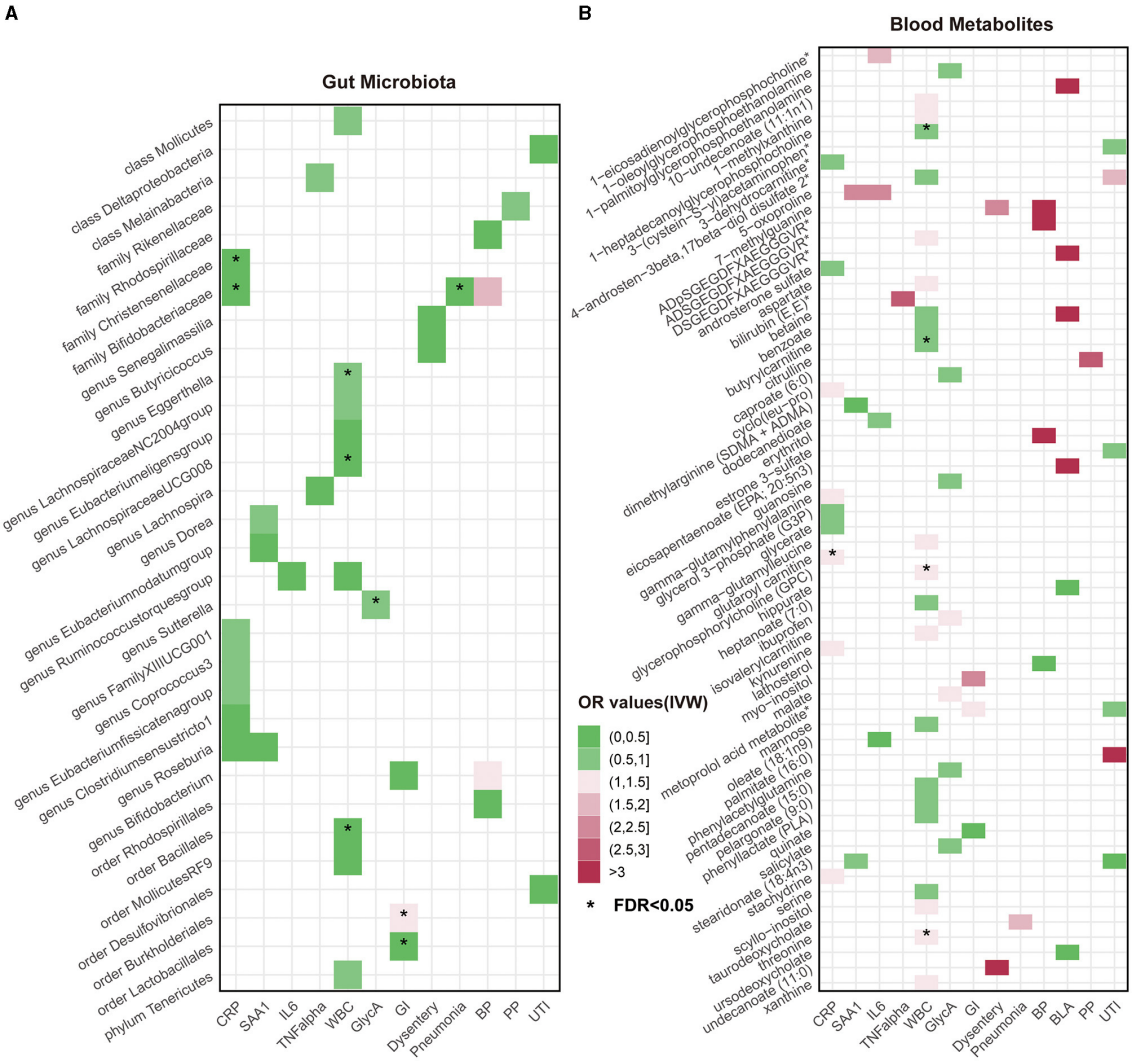
Heatmaps of significant potential causal associations in at least three MR methods including IVW. **(A)** Heatmap of Odds Ratios (ORs) between gut microbiota and all inflammatory factors and risks of seven infections under IVW analysis. The color intensity represents the magnitude of ORs. An asterisk (*) indicates cases where the false discovery rate (*FDR*) value for the IVW method is less than 0.05. **(B)** Heatmap of causal relationship ORs between metabolites and all inflammatory factors and risks of seven infections under the IVW method. CRP, C-reactive protein; SAA1, serum amyloid A1; IL6, interleukin 6; TNF-α, tumor necrosis factor-alpha; WBC, white blood cells; GlycA, glycoprotein acetylation; GI, gastrointestinal infections; BP, bacterial pneumonia; BLA, bronchopneumonia and lung abscess; PP, pneumococcal pneumonia; UTI, urinary tract infections.

**Figure 3:**
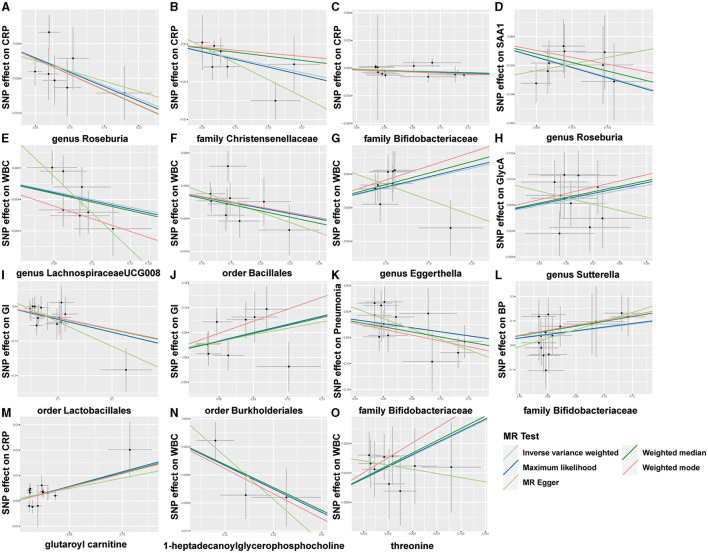
Scatterplots of five MR results. **(A–L)** Scatterplots of the five MR models for 9 gut microbes on CRP, SAA1, WBC, GlycA, GI, pneumonia, and bacterial pneumonia (BP). **(M–O)** Scatterplots of the five MR models for three metabolites on CRP and WBC.

Overall, after multiple-testing correction, nine associations exceeded the strict threshold ([*FDR*] < 0.05, Figure 2). A genetically predicted increase in family *Bifidobacteriaceae* (Beta[95%CI] _IVW_ = −0.034 [−0.055, −0.012]) and *Christensenellaceae* (Beta[95%CI] _IVW_ = −0.038 [−0.062, −0.013]) demonstrated a suggestive inverse association with CRP levels. Genus *Lachnospiraceae* (Beta[95%CI] _IVW_ = −0.033 [−0.052, −0.015]) and order *Bacillales* (Beta[95%CI] _IVW_ = −0.016 [−0.027, −0.006]) were related to decreased WBC; genera *Eggerthella* (Beta[95%CI] _IVW_ = −0.024 [0.009, 0.038]) and *Sutterella* (Beta[95%CI] _IVW_ = 0.098 [0.052, 0.143]) were related to increased WBC and GlycA levels, respectively. As for the infectious risks, family *Bifidobacteriaceae* (Beta[95%CI] _IVW_ = −0.391 [−0.621, −0.161]) and order *Lactobacillales* (Beta[95%CI] _IVW_ = −0.374 [−0.597, −0.151]) were respectively linked to reduced pneumonia and gastrointestinal infection risk, while order *Burkholderiales* (Beta[95%CI] _IVW_ = 0.533 [0.216, 0.850]) were linked to elevated GI risk. Importantly, we also observed three bacterial traits were common to two different inflammatory markers or infectious risks, such as family *Bifidobacteriaceae*, which was negatively associated with both CRP and pneumonia risk, and genus *Roseburia*, which was simultaneously associated adversely with CRP [Beta (95%CI) _IVW_ = −0.040 (−0.066, −0.013), *P*_IVW_ = 0.004] and SAA1 [Beta (95%CI) _IVW_ = −0.280 (−0.465, −0.095), *P*_IVW_ = 0.003] levels. Interestingly, the *Bifidobacteriaceae* family was associated with an increased risk of pneumonia subtype BP [Beta (95%CI) _IVW_ = 0.718 (0.160, 1.276), *P*_IVW_ = 0.117]. The scatterplots of the SNP effect sizes for the MR results are displayed in Figure 3 and Supplementary Figure 2. The sensitivity estimators were relatively consistent across different MR tests, with similar directions and comparable magnitudes of effect.

**Figure 4:**
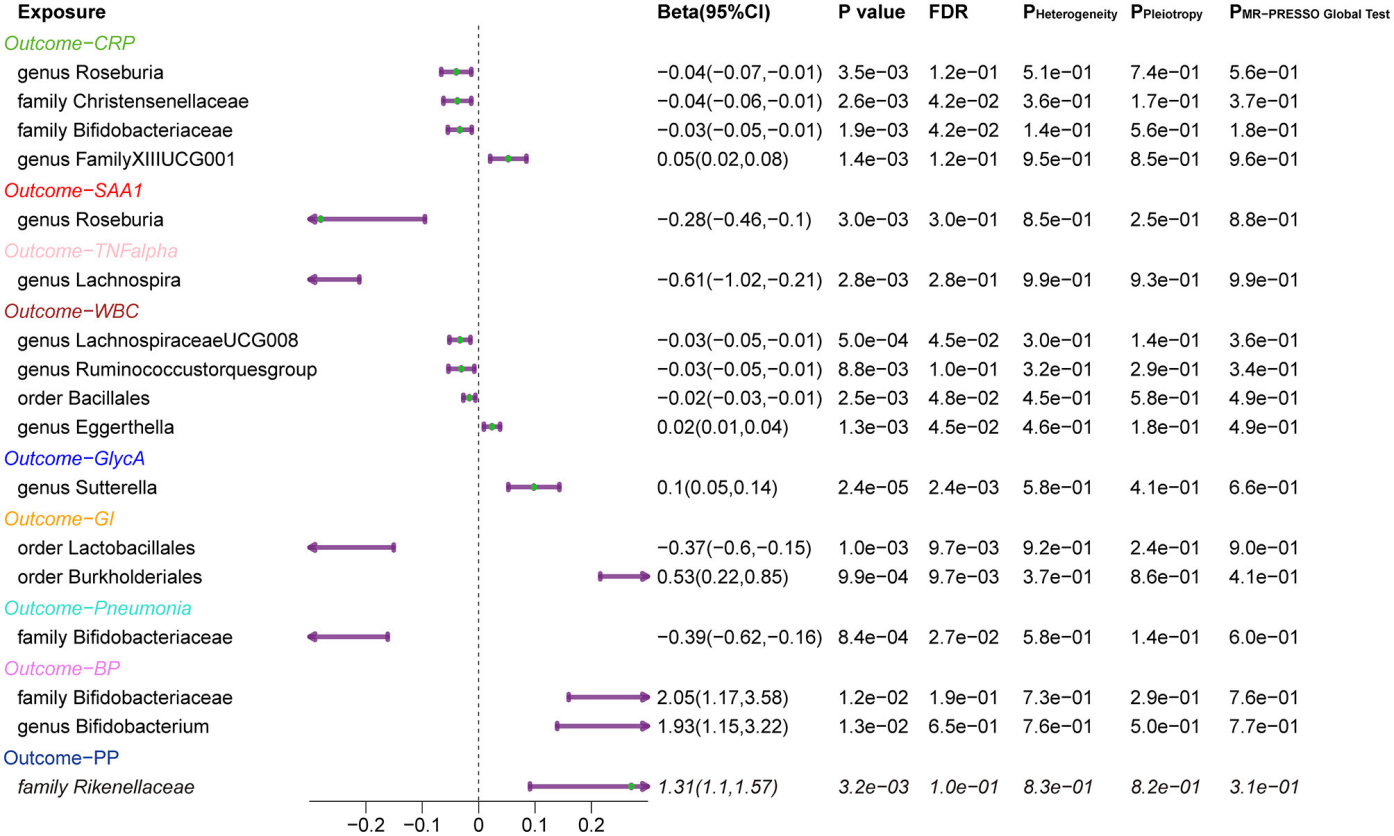
Forest Plots of MR Results for the Identified Causal Relationship between Gut Microbiota and Inflammation and Risks of Infection. 95% CI: 95% confidence interval; P Heterogeneity: *P-*value of heterogeneity test; P Pleiotropy: *P-*value of the intercept of MR Egger; P MR-PRESSO Global test: *P-*value of the MR-PRESSO global test. CRP, C-reactive protein; SAA1, serum amyloid A1; TNF-α, tumor necrosis factor-alpha; WBC, white blood cells; GlycA, glycoprotein acetylation; GI, gastrointestinal infections; BP, bacterial pneumonia; PP, pneumococcal pneumonia.

Finally, a leave-one-out sensitivity analysis was further employed to confirm the reliability and stability of these results (Supplementary Figures 1, 2). The identified causal associations (Figure 4) include four taxa (genus *Roseburia* and family *XIII UCG001*, families *Bifidobacteriaceae*, and *Christensenellaceae*) for CRP, genus *Roseburia* for SAA1, genus *Lachnospira* for TNF-α, four taxa (genera *Eggerthella, Lachnospiraceae*, and *Ruminococcustorques*, order *Bacillales*) for WBC, and genus *Sutterella* for GlycA remains displayed no sensitivity with any single IVs, indicating robust causal links from the identified taxa to the corresponding outcomes. In addition, orders *Burkholderiales* and *Lactobacillales* for GI, family *Bifidobacteriaceae* for pneumonia, family *Bifidobacteriaceae* and genus *Bifidobacterium* both for BP, and family *Rikenellaceae* for PP also passed the leave-one-out analysis.

### Two-sample MR analysis of blood metabolites on inflammation and risk of infections

Utilizing the IVW method, we identified 27, 10, 11, 13, 41, and 18 significant annotated metabolites for CRP, SAA1, IL-6, TNF-α, WBC, and GlycA (*P*_IVW_ < 0.05, Supplementary Table S8). There are 11, 11, 16, 14, 22, 10, and 20 significant annotated metabolites for risk of GI, dysentery, pneumonia, BP, BLA, PP, and UTI. The above associations were further validated by MR-Egger, MR-PRESSO, maximum likelihood, and weighted median methods. The results of all MR methods are shown in Supplementary Table S9. Then, 9, 3, 4, 1, 21, and 7 annotated metabolites displayed significant associations with CRP, SAA1, IL-6, TNF-α, WBC, and GlycA, and 3, 2, 1, 4, 6, 2, and 6 metabolites were significant for the risk of GI, dysentery, pneumonia, BP, BLA, PP, and UTI in at least three MR methods (*P* < 0.05) and demonstrated no heterogeneity or pleiotropy effects (Supplementary Table S10).

As shown in Figure 2, only five associations remained significant after correction for *FDR* ([*FDR*] < 0.05). Glutaroyl carnitine is correlated with elevated CRP levels (Beta [95%CI] _IVW_ = 0.112 [0.057, 0.166]), threonine (Beta [95%CI] _IVW_ = 0.200 [0.093, −0.307]) is correlated with increased WBC, and 1-heptadecanoylglycerophosphocholine (Beta [95%CI] _IVW_ = −0.246 [−0.363, −0.128]) is correlated with decreased WBC. Notably, we observed that two metabolites that were common to two different inflammatory markers or infectious risk, such as 5-oxoproline positively associated with both IL-6 (Beta[95%CI] _IVW_ = 0.697 [0.065, 1.329], *P*_IVW_ = 0.031) and SAA1 (Beta [95%CI] _IVW_ = 0.912 [0.254, 1.569], *P*_IVW_ = 0.007), 7-methylguanine positively related to dysentery (Beta [95%CI] _IVW_ = 0.813 [0.012, 1.614], *P*_IVW_ = 0.047) and BP (Beta [95%CI] _IVW_= 2.099 [0.051, 4.147], *P*_IVW_= 0.045), and stearidonate (18:4n3) negatively related to SAA1 (Beta [95%CI] _IVW_ = −0.468 [−0.930, −0.007], *P*_IVW_ = 0.047) and UTI (Beta [95%CI] _IVW_ = −1.025 [−1.666, −0.385], *P*_IVW_ = 0.002). The scatterplots of the SNP effect sizes for the above associations are shown in Figure 3 and Supplementary Figure 3, demonstrating relatively consistent effect direction and magnitude across methods.

**Figure 5:**
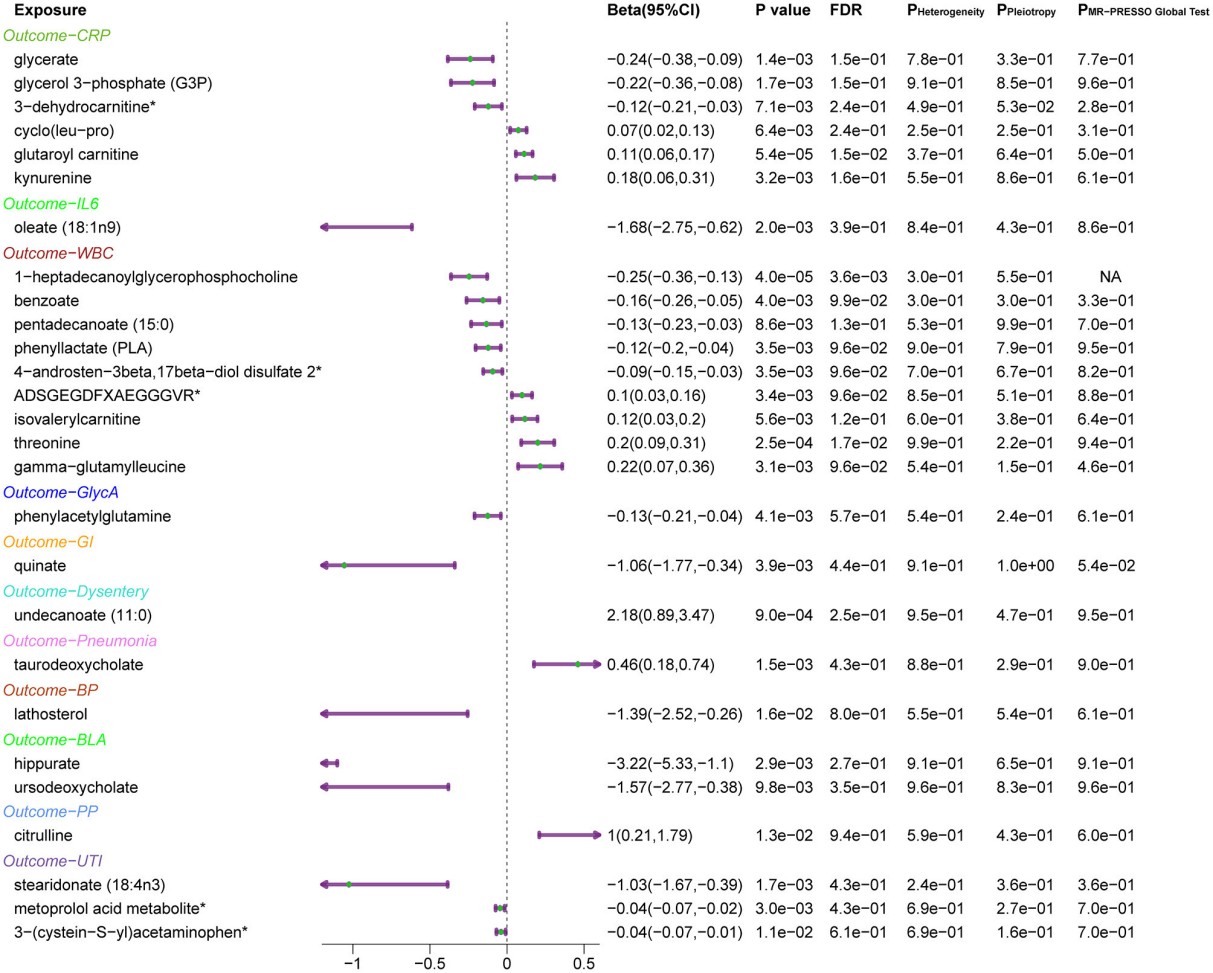
Forest plots of MR results for the identified causal relationship between metabolites and inflammation and risks of infection. The beta value for undecanoate (11:0) exceeds the range of the plot. CRP, C-reactive protein; IL6, interleukin 6; WBC, white blood cells; GlycA, glycoprotein acetylation; GI, gastrointestinal infections; BP, bacterial pneumonia; BLA, bronchopneumonia and lung abscess; PP, pneumococcal pneumonia; UTI, urinary tract infections.

Subsequently, the leave-one-out sensitivity analysis confirmed these identified causal associations (Figure 5), including six metabolites [glycerate, glycerol 3-phosphate, glutaroyl carnitine, kynurenine, cyclo (leu-pro), and 3-dehydrocarnitine] to CRP, oleate (18:1n9) to IL-6, nine metabolites [threonine, pentadecanoate (15:0), benzoate, gamma-glutamylleucine, phenyllactate, ADSGEGDFXAEGGGVR, isovalerylcarnitine, 4-androsten-3beta, 17beta-diol disulfate 2, and 1-heptadecanoylglycerophosphocholine] to WBC, and phenylacetylglutamine to GlycA, with no individual SNPs significantly affecting these associations (Supplementary Figures 1, 4). Besides, quinate to GI, undecanoate (11:0) to dysentery, taurodeoxycholate to pneumonia, lathosterol to BP, two metabolites (hippurate and ursodeoxycholate) to BLA, citrulline to PP, 3-(cysteine-S-yl) acetaminophen, stearidonate (18:4n3), and metoprolol acid metabolite to UTI showed no sensitivity to any single IVs.

### Reverse MR

The results of the reverse MR analysis are shown in Supplementary Tables S11, S12. Causal associations involving fewer than three IVs were not analyzed, using a threshold of *P-*value < 5e-8 for IV selection. Here, WBC has causal effects on order *Bacillales* abundance. Except for this, no significant causal estimates were detected by the five MR methods, suggesting a lack of evidence for a causal effect from the changes in inflammatory markers or infectious risks to identified gut bacterial taxa or blood metabolites.

## Discussion

Systemic inflammation is often accompanied by metabolic alterations and gut microbiota dysbiosis; hence, a comprehensive study to investigate the causal effects of blood metabolomics and the gut microbiome on inflammation and infection risk is of considerable scientific interest. This MR study suggested a potential role of increasing genus *Roseburia* and family *Bifidobacteriaceae* abundance, as well as stearidonate (18:4n3), in mitigating inflammation or infectious risks, which could reduce CRP, SAA1, or GlycA levels, pneumonia, or UTI risk. Conversely, elevating 5-oxoproline levels may enhance inflammatory markers of IL-6 and SAA1 levels. Collectively, these results underscore the importance of elucidating the causal effects between gut microbiota, blood metabolites, inflammatory markers, and infection risk in understanding the biological mechanisms underlying systemic inflammation, which might provide promising targets to ease the monitoring and therapy of inflammation-associated diseases and infectious risks.

The gut microbiota can modulate the host's immune-inflammatory process, thus affecting the development of inflammatory disorders and even infectious risks. This MR study suggested that the genus *Roseburia* may help mitigate CRP and GlycA-indicated inflammation, and the family *Bifidobacteriaceae* may help control CRP levels and pneumonia risk. Consistent with this study, previous studies have also noted an inverse correlation between *Roseburia* and *Bifidobacteriaceae* abundance and CRP levels (Malaguarnera et al., 2012; Groeger et al., 2013; Xu et al., 2021; Bao et al., 2022), and their absence is commonly observed in diverse diseases (Tamanai-Shacoori et al., 2017). *Roseburia*, a major butyrate producer, is one of the most abundant species in the gut microbiota of healthy humans (Aminov et al., 2006). Both *Roseburia* and *Bifidobacteria* are vital for maintaining gut homeostasis and anti-inflammatory effects by promoting butyrate production (Kasahara et al., 2018; Seo et al., 2020). Notably, *Bifidobacteria* show butyrogenic effects through cross-feeding interactions with other butyrate-producing bacteria like *Roseburia* (Riviere et al., 2016). Butyrate has anti-inflammatory properties, can induce Treg cell differentiation, limits pro-inflammatory cytokines by inhibiting the nuclear factor kappa-B (NF-kB) pathway, releases anti-inflammatory molecules, and maintains gut homeostasis by secreting antimicrobial peptides (Tamanai-Shacoori et al., 2017; Singh et al., 2022). Interestingly, *Roseburia intestinalis* interacts with dietary plant polysaccharides and can further alleviate systemic inflammation and atherosclerotic lesions via butyrate production (Kasahara et al., 2018). Therefore, our results support the prospect of shifting the interest from simply increasing *Bifidobacterial* concentrations to stimulating or supplementing butyrate-producing bacteria like *Roseburia* as next-generation candidate probiotics for alleviating inflammation magnitude (Zhang et al., 2022).

*Bifidobacteria* have been shown to interact with human immune cells, involving innate and adaptive immune processes (Ruiz et al., 2017). *Bifidobacteriaceae*, the sole family within *Bifidobacteriales*, encompasses many species with demonstrated multifaceted probiotic effects (Hidalgo-Cantabrana et al., 2017). The typical genus, *Bifidobacterium*, has been widely used as a probiotic to maintain gut flora balance and address gastrointestinal disorders (Chen et al., 2021). Extensive research has confirmed that some strains of *Bifidobacteria* could impart anti-inflammatory benefits by inhibiting NF-κB activation and lipopolysaccharide production, reducing IL-1β levels, regulating immune balance and inflammatory response, and countering neutrophil migration (Chen et al., 2021). Decreased *Bifidobacteriaceae* abundance was also observed in children with mycoplasma pneumoniae (Shi et al., 2022) and respiratory tract infection (Li et al., 2019). Existing studies have revealed that intestinal probiotics enhance the host's resistance to pneumonia, and novel therapeutic strategies could exploit the gut-lung axis in bacterial infections (Schuijt et al., 2016). As known, multiple studies have demonstrated that long-term use of probiotics such as *Lactobacillus* or *Bifidobacterium* could significantly reduce the risk of infections, including respiratory and GI (Hojsak et al., 2010; Wolvers et al., 2010; Ozen et al., 2015). Similarly, our results also supported that order *Lactobacillales* were linked to reduced GI risk.

In addition, several potentially harmful bacteria were found to enhance inflammation or infectious risks. We found that order *Burkholderiales* were linked to elevated GI risk. *Burkholderia* are known as mammalian pathogens and consist mainly of pathogenic bacteria (e.g., *Bordetella, Ralstonia*, and *Oxalobacter*). It was already identified as an important pathogen for chronic infections (Lewis and Torres, 2016), including GI (Sanchez-Villamil et al., 2022). Recent studies revealed that type 6 secretion system-dependent blockage of TNF-α signaling and BicA as a *Burkholderia pseudomallei* pathogenesis during GI (Sanchez-Villamil et al., 2022). Besides, the genera *Eggerthella* and *Sutterella* were related to increased WBC and GlycA levels after multiple-testing correction, respectively. It was reported that both genera *Eggerthella* (Chang and Choi, 2023) and *Sutterella* (Hiippala et al., 2016; Kaakoush, 2020) have pro-inflammatory properties. An increasing number of studies have shown that *Eggerthella lenta* could be an important pathogen for humans, even causing life-threatening infection under certain conditions (Jiang et al., 2021b), which can drive Th17 activation in immune-related diseases (Alexander et al., 2022). Although *Sutterella* may have an immunomodulatory role and has been frequently identified as being associated with autism and inflammatory bowel disease (Hiippala et al., 2016), *Sutterella* is a controversial bacterium; whether *Sutterella* species represents the cause or consequence of inflammation and infection remains unclear.

Furthermore, metabolic balance is closely related to immune-inflammatory processes. Specifically, glutaroyl carnitine (C5DC) was the only metabolite that remained significant on elevated CRP levels after *FDR* correction in the IVW analysis. In line with us, C5DC levels were positively correlated with the inflammatory marker IL-1β (Guerreiro et al., 2021). Increased C5DC levels are linked to cardiovascular risks (Zhao et al., 2020) and aging (Carlsson et al., 2021), both of which are commonly accompanied by prolonged chronic inflammation (Shi et al., 2021). Notably, C5DC elevations caused by glutaryl-CoA dehydrogenase deficiency are generally seen in the inborn glutaric aciduria type I disorder, leading to neurological dysfunction and high inflammatory states (Zhao et al., 2014). In addition, after *FDR* correction, 1-heptadecanoyglycerophosphocholine decreased WBC levels, and threonine increased WBC levels, still statistically significant. The results in aged mice showed that ingestion of α-glycerophosphocholine (GPC) decreased the expression levels of aging-related long-term enhancement genes associated with long-term enhancement of gene expression levels (Narukawa et al., 2020). There is growing evidence that there is a strong association between leukocyte levels of telomeres and aging (Aviv, 2004). Threonine is used by lymphocytes to increase antibody secretion to maintain immune function (Tang et al., 2021). The other three metabolites are also worth mentioning, which showed similar causal effects on two inflammatory makers or infectious phenotypes. 5-oxoproline is a well-known mediator inducing inflammation and oxidative stress (Pederzolli et al., 2010; Van Der Pol et al., 2018), which could be biomarkers for early diagnosis of sepsis, reflecting an imbalance in glutamine and glutathione metabolism (Lu et al., 2022). 7-methylguanine increases the risk of both dysentery and hypertension. Stearidonate (18:4n3) is a specific omega-3 polyunsaturated fatty acid (PUFA) that is abundant in seafood and is related to reduced SAA1 levels and the risk of urinary tract infection. In the past few decades, many epidemiological studies have been reported on the myriad health benefits of omega-3 PUFAs (Shahidi and Ambigaipalan, 2018), including preventing and resolving inflammation disorders (Yates et al., 2014). Collectively, these findings indicate the potential for interventions modulating the levels of these metabolites to impact the body's immune inflammatory response, further highlighting the therapeutic potential of metabolic modulation.

This study used the largest publicly available GWAS statistics on the gut microbiome and blood metabolome to conduct MR analysis, which can minimize the effects of potential confounders and enhance the causal inference in the associations (Davies et al., 2018). Strict quality control procedures and sensitivity analysis approaches were used to ensure the robustness of the MR estimates. Hence, the associations identified in this study could help guide further exploration into the mechanisms linking the gut microbiome, metabolic signature, and inflammatory and infectious phenotypes. Importantly, our results provide new insights into the potential of supplementing probiotics like *Roseburia* and *Bifidobacteriaceae* and metabolic reprogramming strategies for alleviating inflammation and infectious risks. Furthermore, we could extend the potential benefits of gut microbiota to systemic inflammatory diseases beyond the gut. Although current research is still limited, this study could help us improve our knowledge on the molecular mechanism behind systemic inflammation, suggesting potential therapeutic targets. Nonetheless, this study has certain limitations. First, though MR methods enable causal inference from exposure to the outcome, the magnitude of the effects is hard to estimate accurately. Further large-scale clinical trials and mechanistic research are required. Second, this study focused on populations of European descent without extrapolating the results to other ethnic groups. Thirdly, data on bacterial taxa at the species level was unavailable; further study is needed to illustrate the causal links between the specific species or strains and inflammatory phenotypes. Additionally, it is important to note that the GWAS data on gut microbiota did not take into account the potential impact of medication treatments, such as antibiotics, on an individual's gut microbiota balance. This highlights the necessity for further experimental validation. Furthermore, our study does not account for the duration of inflammation or the timing of blood sample collection, which could significantly affect biomarker levels.

## Conclusion

This study provides novel evidence for a causal association between metabolites, gut microbiota, inflammation, and the risk of infection. Our MR study suggests a potentially protective effect of the genus *Roseburia*, family *Bifidobacteriaceae*, and stearidonate (18:4n3) against inflammation or infectious risks. In contrast, 5-oxoproline showed a possible pro-inflammatory role. These findings shed light on new insights into metabolic and microbially mediated alterations in inflammatory extent, suggesting their potential implications in the prevention and treatment of inflammation and infections.

## Data availability statement

Publicly available datasets were analyzed in this study. The methodology section of this article details the data sources.

## Author contributions

YL: Writing—review & editing, Writing—original draft, Visualization, Software, Methodology, Data curation, Conceptualization. QZ: Conceptualization, Software, Writing—original draft, Writing—review & editing. GG: Writing—review & editing, Software, Data curation. ZX: Formal analysis, Investigation, Writing—review & editing. SL: Writing—review & editing, Software, Data curation, Conceptualization. CL: Writing—review & editing, Software, Data curation. YW: Methodology, Software, Writing—review & editing. LW: Software, Writing—review & editing, Methodology. SZ: Writing—review & editing.
