# Supplementary material for: Causal associations of genetically predicted gut microbiota and blood metabolites with inflammatory states and risk of infections: a Mendelian randomization analysis
Source: Front Microbiol. 2024 Mar 22;15:1342653. doi: 10.3389/fmicb.2024.1342653 (PMC10995310; doi:10.3389/fmicb.2024.1342653)

# Supplementary Materials

**Supplementary Figure 1. Forest Plots of "Leave-One-Out" Analysis for Causal Relationships between Roseburia, Bifidobacteriaceae and Inflammation and Risk of Infections, as well as Other Causal Associations under IVW Analysis with FDR < 0.05.**


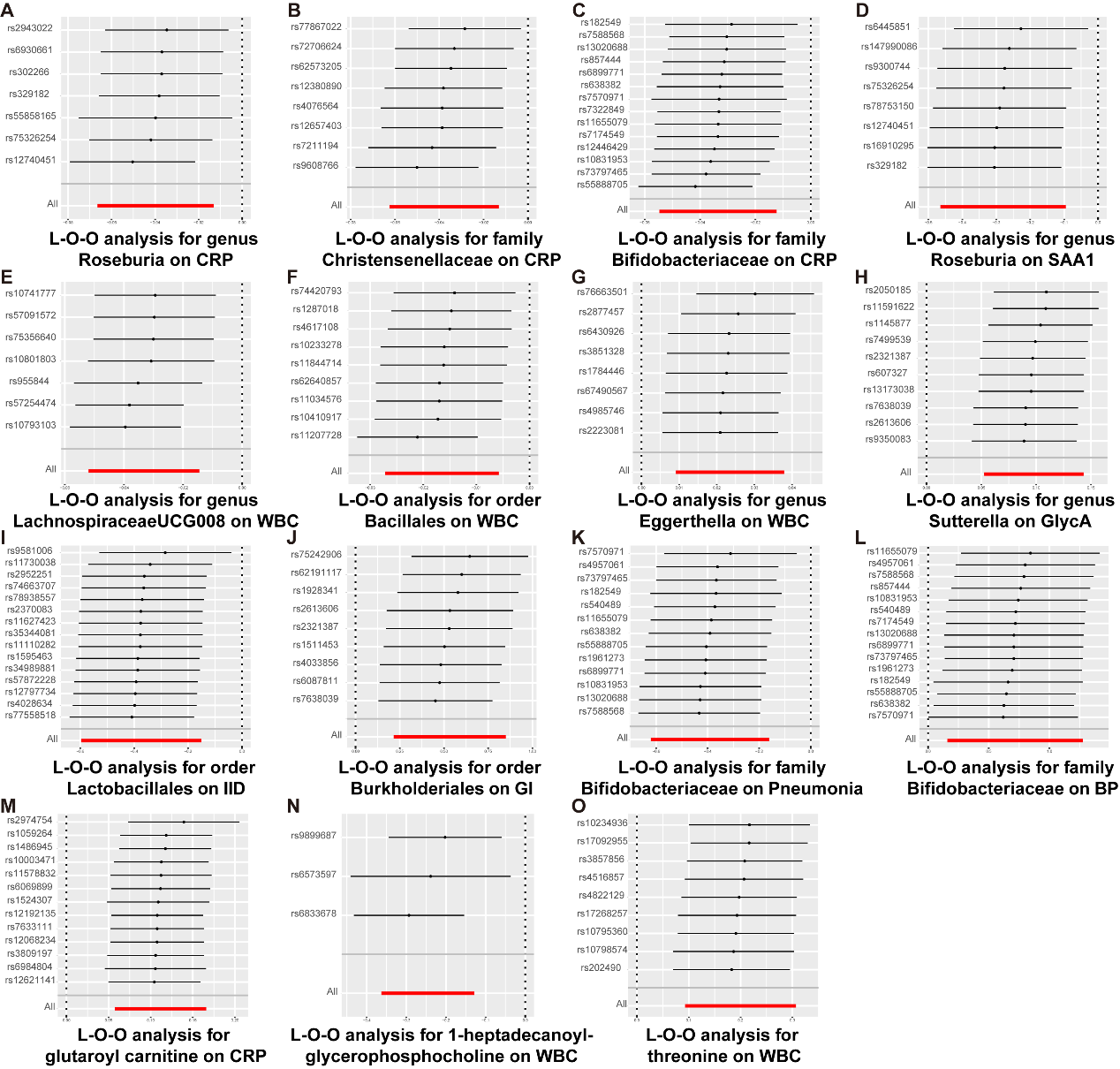


(A-L) Forest plots of the MR model for 9 gut microbes on CRP, SAA1, WBC, GlycA, GI, pneumonia and bacterial pneumonia using "Leave-One-Out" method. (M-O) Forest plots of the MR model for 3 metabolites on CRP and WBC using "Leave-One-Out" method.

**Supplementary Figure 2. Scatter Plots and "Leave-One-Out" (LOO) Analysis of Causal Associations between Gut Microbiota (Excluding those result shown in Figure 3) and Inflammatory Factors and Risks of Infections.**


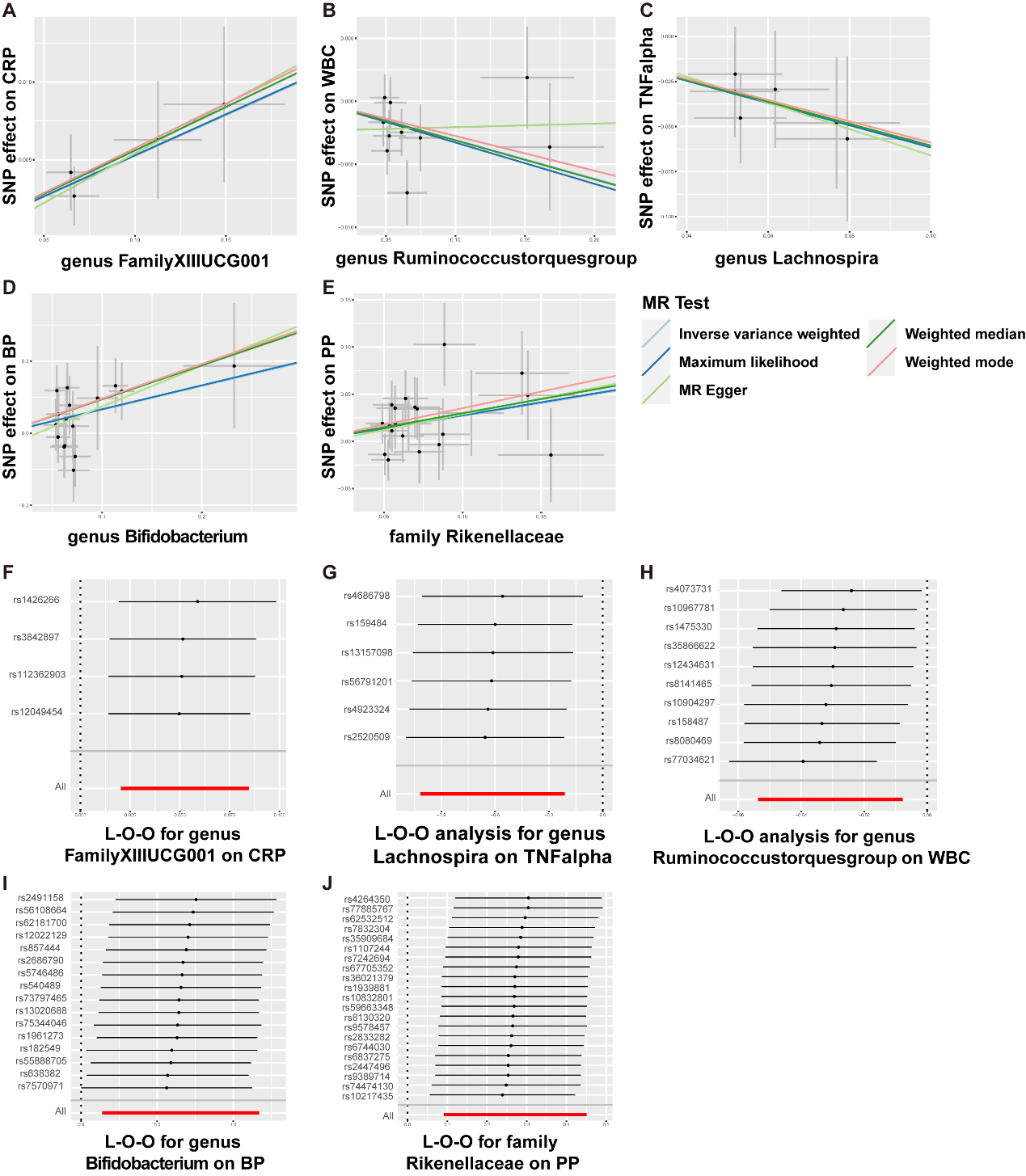


**Supplementary Figure 3. Scatter Plots of Causal Associations between Metabolites (Excluding those result shown in Figure 3) and Inflammatory Factors and Risks of Infections.**


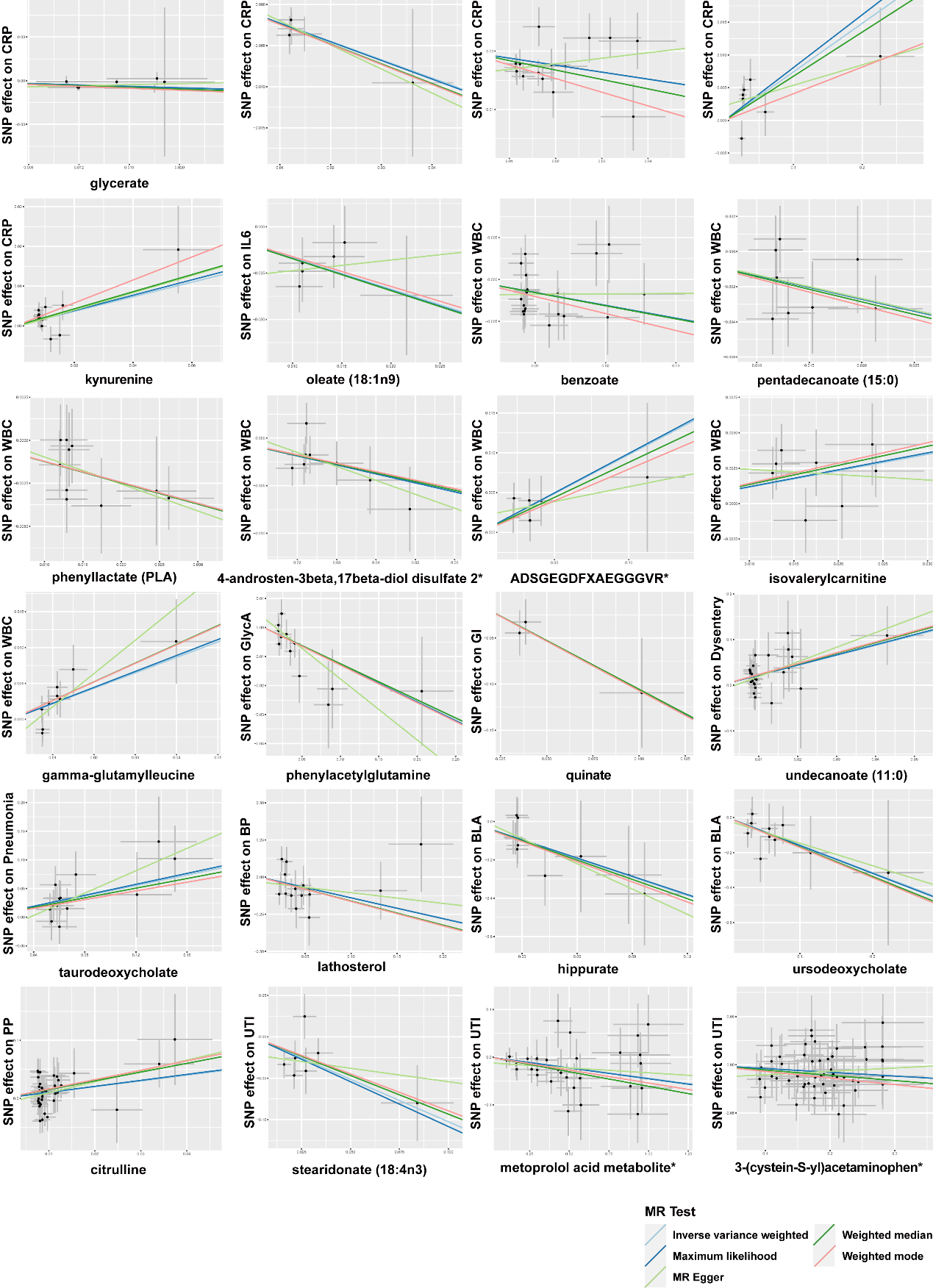


**Supplementary Figure 4. Forest Plots of "Leave-One-Out" Analysis for Causal Associations between Metabolites (Excluding those result shown in Figure 3) and Inflammatory Factors and Risk of Infections.**


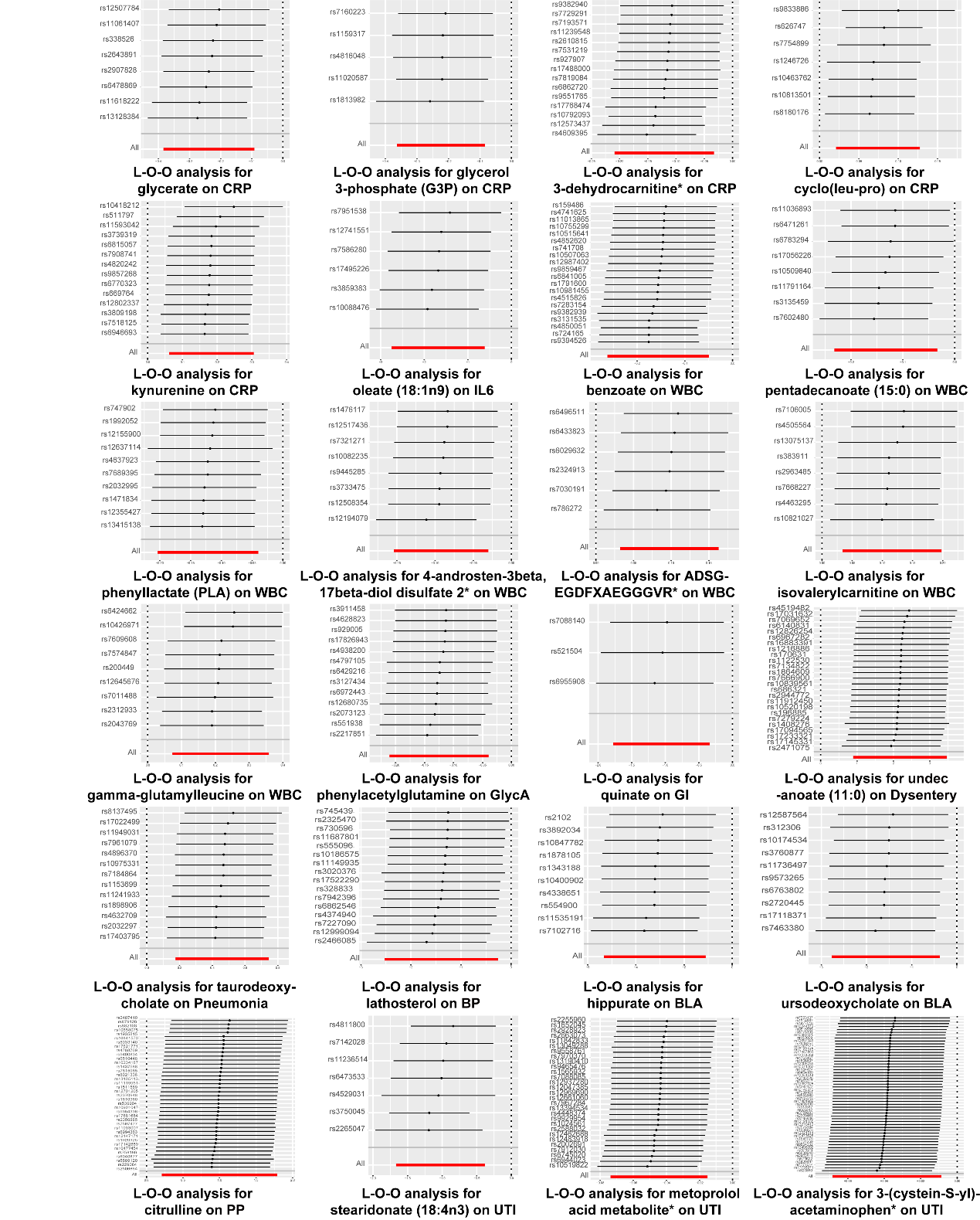

Supplement: Supplementary file 1 [file Data_Sheet_1.zip › Table1/Supplementary Figures.docx]
